# Novel insights into post-marketing adverse events associated with lenvatinib: A comprehensive analysis utilizing the FAERS database

**DOI:** 10.1016/j.heliyon.2024.e28132

**Published:** 2024-03-13

**Authors:** Zhe Yu, Jing Luo, Hongshan Wei

**Affiliations:** a Peking University Ditan Teaching Hospital, Beijing, 100015, China; b Department of Gastroenterology, Beijing Ditan Hospital, Capital Medical University, Beijing, 100015, China

**Keywords:** lenvatinib, ROR, PRR, BCPNN, EBGM

## Abstract

**Purpose:**

The primary aim of this study was to closely monitor and identify adverse events (AEs) linked to lenvatinib, a pharmacotherapeutic agent employed for the management of renal cell carcinoma, thyroid cancer, and hepatocellular carcinoma. The ultimate goal was to optimize patient safety and provide evidence-based guidance for the appropriate utilization of this medication.

**Methods:**

A comprehensive collection and analysis of reports from the FDA Adverse Event Reporting System (FAERS) database was conducted, encompassing the period from the first quarter of 2015 to the first quarter of 2023. Disproportionality analysis, employing robust algorithms including ROR, PRR, BCPNN, and EBGM was employed for effective data mining to quantify signals associated with lenvatinib-related AEs.

**Results:**

Among the collected reports, a total of 15,193 cases were identified where lenvatinib was the "primary suspected (PS)" drug, resulting in 50,508 lenvatinib-induced AEs. An analysis was conducted to examine the occurrence of lenvatinib-induced adverse drug reactions (ADRs) across 26 organ systems. The findings revealed the presence of expected ADRs, including diarrhea, vomiting, stomatitis, hepatic encephalopathy, decreased appetite, dehydration, decreased weight, and electrolyte imbalances, which were consistent with the information provided in the drug labels. Furthermore, unexpected significant ADRs were observed at the preferred terms (PT) level, such as interstitial lung disease, pneumothorax, hypophysitis, failure to thrive, polycythemia, hypopituitarism, spontaneous pneumothorax, pulmonary cavitation, and limbic encephalitis. These findings indicated the potential occurrence of adverse effects that are currently not documented in the drug instructions.

**Conclusions:**

This study has successfully detected novel and unforeseen signals pertaining to ADRs associated with the administration of lenvatinib, thereby contributing significant insights into the intricate correlation between ADRs and the utilization of lenvatinib. The outcomes of this investigation underscore the utmost significance of continuous monitoring and vigilant surveillance in order to promptly identify and effectively manage AEs, consequently enhancing overall patient safety and well-being in the context of lenvatinib therapy.

## 1 Introduction

Lenvatinib, a multi-target kinase inhibitor, has gained approval from the US Food and Drug Administration (FDA) for the treatment of renal cell carcinoma, thyroid cancer, hepatocellular carcinoma, and endometrial carcinoma. Specifically, lenvatinib received FDA approval for thyroid cancer in 2015, renal cell carcinoma in 2016, hepatocellular carcinoma in 2018, and endometrial carcinoma in 2019. These diseases exhibit a significant global burden, with renal cell carcinoma affecting approximately 2.2 million individuals worldwide annually, thyroid cancer impacting around 60,000 individuals in the United States annually, hepatocellular carcinoma ranking as the third leading cause of cancer-related mortality worldwide, and the overall incidence of endometrial carcinoma has risen by 132% in the last 30 years [[1], [2], [3], [4]].

The mechanism of action of lenvatinib entails the inhibition of multiple kinases, which encompass vascular endothelial growth factor receptors (VEGFR-1, 2, and 3), platelet-derived growth factor receptor beta (PDGFR-β), fibroblast growth factor receptors (FGFR-1 and 4), and fibroblast growth factor receptor 2 (FGFR-2), among others. These kinases play pivotal roles in tumor growth, angiogenesis, and metastasis [5]. The widespread use of lenvatinib in clinical settings and its manageable toxicity levels suggest the importance of monitoring adverse events (AEs) in real-world data. Meanwhile, there have been case reports indicating rare adverse reactions associated with the use of lenvatinib either as a monotherapy or in combination with other drugs, highlighting a discrepancy between the current high utilization rate of lenvatinib and our understanding of its safety profile [[6], [7], [8]]. Therefore, it is important to study and analyze the safety profile of lenvatinib in real-world settings.

The FDA Adverse Event Reporting System (FAERS) is a database that contains information on AEs associated with the use of drugs. It can be used to identify potential drug safety signals and to assess the safety of drugs. Several studies have demonstrated the feasibility and usefulness of FAERS for monitoring AEs associated with the use of drugs in real-world settings. Illustratively, in Yang's investigation, the application of data mining methodologies on the FAERS database revealed hitherto unreported adverse reactions, including eye disorders, associated with topotecan [9]. In a parallel vein, Shu's scholarly inquiry harnessed the potential of data mining techniques on the FAERS database to unveil previously unanticipated AEs linked specifically to risankizumab [10]. Consequently, the adoption of the FAERS database as a pivotal tool for conducting real-world safety evaluations of lenvatinib demonstrates exceptional feasibility and bears profound clinical implications.

Our study leveraged a comprehensive disproportionality analysis of adverse reactions related to lenvatinib in real-world data extracted from the FAERS database, employing a robust selection of four distinct algorithms: Reporting Odds Ratio (ROR), Proportional Reporting Ratio (PRR), Bayesian Confidence Propagation Neural Network (BCPNN), and Empirical Bayesian Geometric Mean (EBGM). This methodological approach served as a robust mechanism for surveying the efficacy and safety of lenvatinib treatment by scrutinizing the strength of signals within real-world data, thereby facilitating the efficient detection and management of AEs. Ultimately, our research endeavors aimed to augment patient safety during the course of lenvatinib therapy by promoting a more comprehensive and effective AE monitoring and management framework.

## 2 Materials and methods

### 2.1 Data and preprocess

This research study undertook an analysis of AEs data related to lenvatinib in the FAERS database. Data collection and preprocessing were performed using Statistical Analysis System (SAS) 9.4 software. After thoroughly cleansing and standardizing the data, any duplicate cases were removed. To identify adverse drug reactions (ADRs), statistical methods were employed. This study collected AEs related to lenvatinib over past 8 years, and the AEs were classified into preferred terms (PTs) and system organ classes (SOCs) that reflect different levels of the Medical Dictionary for Regulatory Activities (MedDRA). The standardization status of the drug name for lenvatinib is presented in Supplementary table S1.

Our research specifically targeted lenvatinib as the primary suspect (PS) drug in our pursuit of identifying ADRs within the FAERS database. Furthermore, we employed a classification to categorize severe clinical outcomes, which encompassed events such as death, disability, hospitalization, and life-threatening circumstances. Additionally, we examined other factors, including gender, age, and reporting country, in order to gain a more comprehensive understanding of the data.

### 2.2 Statistical analysis

To identify potentially disproportionate reporting of AEs associated with the usage of lenvatinib, our study adopted a comprehensive array of algorithms. Specifically, four distinct algorithms, namely ROR, PRR, BCPNN, and EBGM, were employed to discern significant ADRs by analyzing reported AEs comprehensively [[10], [11], [12]]. Disproportionality analysis can define relative risk and positive signals by calculating the significance of "drug-adverse event" within the "background", while the combined use of these four algorithms has to some extent minimized the false positive rate. However, in order to accurately calculate these four metrics, a set of values (denoted as a, b, c, and d) corresponding to target and non-target AEs following lenvatinib use and non-lenvatinib use must be obtained beforehand (refer to Supplementary table S2). The precise equations for each of the four algorithms are expounded explicitly in Supplementary table S3.

## 3 Results

### 3.1 Real-world population characteristics

The FAERS database, following the elimination of replicated reports, documented 11, 486, 919 reports from January 2015 to March 2023. There were 15,193 reports with lenvatinib as the PS. And a total of 50,508 AEs were detected when lenvatinib was considered the PS drug. Table 1 provides a comprehensive description of the clinical characteristics associated with lenvatinib-related events.

**Table 1:** The characters of case reports associated with lenvatinib as primary suspected drug from 2015 Q1 to 2023 Q1.

	Lenvatinib	
Number of events	Counts, n = 15193	Percentage
Gender		
Male	7505	49.40%
Female	7073	46.55%
Unknown	615	4.05%
**Age**		
< 18	100	0.66%
≥18, < 45	405	2.67%
≥45, < 65	4351	28.64%
≥65, < 75	4401	28.97%
75≤	2702	17.78%
Unknown	3234	21.29%
**Reported Countries (the top ranked)**		
Japan	4897	32.23%
United States of America	3802	25.02%
France	287	1.89%
China	286	1.88%
Spain	218	1.43%
**Serious Outcomes**		
Death	2558	16.84%
Disability	178	1.17%
Hospitalization	11096	73.03%
Life-threatening	706	4.65%
**Etiology**		
Hepatocellular Carcinoma	4109	27.05%
Endometrial Cancer	2388	15.72%
Thyroid Cancer	1969	12.96%
Renal Cell Carcinoma	1093	7.19%
Renal Cancer	693	4.56%
Hepatic Cancer	566	3.73%
Used For Unknown Indication	427	2.81%
Papillary Thyroid Cancer	400	2.63%
Clear Cell Renal Cell Carcinoma	372	2.45%
Anaplastic Thyroid Cancer	346	2.28%

The gender distribution in case reports documenting all AEs was found to be almost equal between males (49.40%) and females (46.55%). Of the cases where patient age was recorded, those aged between 65 and 75 years old had a higher likelihood of experiencing AEs compared to other age groups. This age range accounted for 28.97% (4401) of cases. We also conducted an analysis to determine the countries with the highest number of reported AEs related to lenvatinib. The findings revealed that Japan accounted for the highest proportion, with 4897 reports, representing approximately 32.23% of the total. The United States followed closely with 3802 reports, accounting for approximately 25.02%. Additionally, France, China, and Spain demonstrated a lower presence, with 287 (1.89%), 286 (1.88%), and 218 (1.43%) reports, respectively. Hospitalization accounted for the highest proportion of severe AE outcomes (11096, 73.03%), followed by death (2558, 16.84%), life-threatening events (706, 4.65%), and disability (38, 1.17%). Furthermore, among the indications for the use of lenvatinib, hepatocellular carcinoma (4109, 27.05%), endometrial cancer (2388, 15.72%), thyroid cancer (1969, 12.96%), and renal cell carcinoma (1093, 7.19%) ranked as the top four, which is consistent with the FDA-approved indications.

### 3.2 Detection of signal strength at SOC level

At the SOC level, the signal strength and reporting frequency of lenvatinib are presented in Table 2. Statistical analysis revealed that lenvatinib-induced AEs occurred in 26 targeted SOCs. Among the four algorithms analyzed, at least one significant SOC was identified as meeting the criteria, including gastrointestinal disorders (SOC: 10,017,947, 9798), investigations (SOC: 10,022,891, 5199), nervous system disorders (SOC: 10,029,205, 3901), metabolism and nutrition disorders (SOC: 10,027,433, 3679), respiratory, thoracic and mediastinal disorders (SOC: 10,038,738, 3105), vascular disorders (SOC: 10,047,065, 2812), renal and urinary disorders (SOC: 10,038,359, 1689), neoplasms benign, malignant and unspecified (including cysts and polyps) (SOC: 10,029,104, 1563), Hepatobiliary disorders (SOC: 10,019,805, 1415), Cardiac disorders (SOC: 10,007,541, 1175), Endocrine disorders (SOC: 10,014,698, 730), Congenital, familial and genetic disorders (SOC: 10,010,331, 26).

**Table 2:** The signal strength of AEs of Lenvatinib at the SOC level in the FAERS database.

SOC Name	SOC Code	Case Numbers	ROR (95% CI)	PRR (95% CI)	Chi square	IC (95% CI)	EBGM (95% CI)
Gastrointestinal disorders	10,017,947	9798	2.79 (2.73–2.85)^a^	2.44 (2.40–2.49)^a^	9054.14^a^	1.29 (1.25–1.32)^a^	2.44 (2.39–2.49)^a^
General disorders and administration site conditions	10,018,065	6021	0.77 (0.74–0.79)	0.79 (0.77–0.81)	382.05	−0.33 (-0.37–0.29)	0.79 (0.77–0.81)
Investigations	10,022,891	5199	2.25 (2.19–2.32)^a^	2.13 (2.07–2.18)^a^	3246.79^a^	1.09 (1.04–1.13)^a^	2.12 (2.06–2.18)^a^
Nervous system disorders	10,029,205	3901	1.09 (1.06–1.13)^a^	1.08 (1.05–1.12)^a^	27.27	0.12 (0.07–0.16)^a^	1.08 (1.05–1.12)
Metabolism and nutrition disorders	10,027,433	3679	4.16 (4.02–4.30)^a^	3.93 (3.81–4.05)^a^	8133.61^a^	1.97 (1.92–2.02)^a^	3.91 (3.78–4.04)^a^
Respiratory, thoracic and mediastinal disorders	10,038,738	3105	1.46 (1.41–1.52)^a^	1.44 (1.39–1.49)^a^	428.74	0.52 (0.47–0.57)^a^	1.44 (1.38–1.49)
Vascular disorders	10,047,065	2812	3.20 (3.08–3.32)^a^	3.07 (2.96–3.19)^a^	3986.31^a^	1.62 (1.56–1.67)^a^	3.06 (2.95–3.18)^a^
Infections and infestations	10,021,881	2257	0.98 (0.94–1.03)	0.99 (0.95–1.03)	0.54	−0.02 (-0.08-0.04)	0.99 (0.94–1.03)
Musculoskeletal and connective tissue disorders	10,028,395	2037	0.86 (0.82–0.90)	0.86 (0.83–0.90)	45.55	−0.21 (-0.28–0.15)	0.86 (0.83–0.9)
Skin and subcutaneous tissue disorders	10,040,785	1955	0.73 (0.70–0.76)	0.74 (0.71–0.77)	187.93	−0.43 (-0.5–0.37)	0.74 (0.71–0.77)
Renal and urinary disorders	10,038,359	1689	1.82 (1.73–1.91)^a^	1.79 (1.71–1.88)^a^	599.14	0.84 (0.77–0.91)^a^	1.79 (1.7–1.88)
Neoplasms benign, malignant and unspecified	10,029,104	1563	1.71 (1.63–1.80)^a^	1.69 (1.61–1.78)^a^	448.90	0.76 (0.68–0.83)^a^	1.69 (1.61–1.78)
Hepatobiliary disorders	10,019,805	1415	3.90 (3.70–4.12)^a^	3.82 (3.63–4.02)^a^	2953.10^a^	1.93 (1.85–2)^a^	3.81 (3.61–4.01)^a^
Cardiac disorders	10,007,541	1175	1.16 (1.10–1.23)^a^	1.16 (1.10–1.23)^a^	26.18	0.21 (0.13–0.3)^a^	1.16 (1.09–1.23)
Injury, poisoning and procedural complications	10,022,117	911	0.18 (0.17–0.20)	0.20 (0.19–0.21)	3241.18	−2.33 (-2.43–2.23)	0.2 (0.19–0.21)
Psychiatric disorders	10,037,175	861	0.43 (0.40–0.46)	0.44 (0.41–0.47)	629.64	−1.18 (-1.27–1.08)	0.44 (0.41–0.47)
Endocrine disorders	10,014,698	730	8.11 (7.54–8.73)^a^	8.01 (7.45–8.61)^a^	4432.54^a^	2.99 (2.86–3.08)^a^	7.93 (7.36–8.53)^a^
Blood and lymphatic system disorders	10,005,329	666	0.94 (0.87–1.01)	0.94 (0.87–1.01)	2.61	−0.09 (-0.2-0.02)	0.94 (0.87–1.01)
Eye disorders	10,015,919	231	0.29 (0.26–0.33)	0.30 (0.26–0.34)	389.19	−1.75 (-1.93–1.55)	0.3 (0.26–0.34)
Reproductive system and breast disorders	10,038,604	142	0.52 (0.44–0.62)	0.53 (0.45–0.62)	61.14	−0.93 (-1.16–0.68)	0.53 (0.45–0.62)
Immune system disorders	10,021,428	101	0.20 (0.16–0.24)	0.20 (0.16–0.24)	325.52	−2.32 (-2.59–2.02)	0.2 (0.17–0.24)
Ear and labyrinth disorders	10,013,993	71	0.37 (0.30–0.47)	0.37 (0.30–0.47)	74.95	−1.42 (-1.75–1.07)	0.37 (0.3–0.47)
Surgical and medical procedures	10,042,613	66	0.17 (0.13–0.21)	0.17 (0.13–0.22)	271.70	−2.56 (-2.9–2.19)	0.17 (0.13–0.22)
Social circumstances	10,041,244	64	0.47 (0.37–0.60)	0.47 (0.37–0.60)	37.84	−1.08 (-1.43–0.71)	0.47 (0.37–0.6)
Product issues	10,077,536	33	0.09 (0.06–0.12)	0.09 (0.06–0.12)	320.26	−3.53 (-3.98–2.99)	0.09 (0.06–0.12)
Congenital, familial and genetic disorders	10,010,331	26	2.70 (1.84–3.98)^a^	2.70 (1.84–3.97)^a^	27.79^a^	1.43 (0.79–1.9)^a^	2.7 (1.83–3.96)

a Indicates statistically significant signals in algorithm.

### 3.3 Signal detects at PT level

After setting the standard of meeting all four algorithms simultaneously, a total of 269 lenvatinib-induced AE signals were detected across 20 SOCs. The number of reported preferred terms (PTs) is presented in Supplementary table S4. Lenvatinib's label contains extensive descriptions of AEs, such as hypertension, cardiac dysfunction, arterial thromboembolic events, proteinuria, diarrhea, fistula formation and gastrointestinal perforation, hemorrhagic events, among others. Most of the relevant PTs identified in our study corresponded to these descriptions. It is worth noting that our data mining efforts identified several significant AEs not reported on the drug label, such as interstitial lung disease (PT: 10,022,611), pneumothorax (PT: 10,035,759), hypophysitis (PT: 10,062,767), failure to thrive (PT: 10,016,165), polycythemia (PT: 10,036,051), hypopituitarism (PT: 10,021,067), spontaneous pneumothorax (PT: 10,035,763), pulmonary cavitation (PT: 10,051,738), and limbic encephalitis (PT: 10,078,012). Meanwhile, several PTs listed on the drug label, such as rash (PT: 10,037,844), dizziness (PT: 10,013,573), cough (PT: 10,011,224), insomnia (PT: 10,022,437), dysgeusia (PT: 10,013,911), alopecia (PT: 10,001,760), and hepatotoxicity (PT: 10,019,851) did not meet the criteria for at least one of the four algorithms.

### 3.4 Time to onset of lenvatinib-associated AEs

**Fig. 1:**
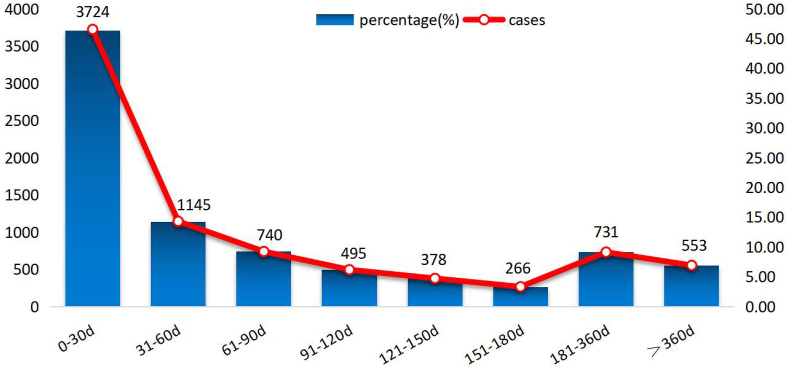
Time to onset of lenvatinib-related AEs.

After excluding reports with inaccurate or missing disease onset times, a total of 8032 AE cases reported onset times, with a median onset time of 37 days (interquartile range [IQR] 10–116 days). As shown in Fig. 1, the majority of lenvatinib-related AEs occurred within the first month (n = 3724, 46.36%), second month (n = 1145, 14.26%), third month (n = 740, 9.21%), and fourth month (n = 495, 6.16%) after initiation of treatment with lenvatinib. Approximately 6.88% of AE events occurred one year or more after treatment.

## 4 Discussion

This study represents the first extensive and systematic pharmacovigilance investigation utilizing the FAERS database to analyze post-marketing AEs linked to lenvatinib. The primary objective of this study was to provide a meticulous and comprehensive characterization, description, and analysis of the reported lenvatinib-related AEs to date. The findings presented herein offer valuable and precise insights into the safety profile of lenvatinib in real-world clinical settings.

In this study, lenvatinib exhibited a higher proportion of AEs in patients aged >45 years (75.39%) with a mean age of 67.2 years. This could be partly attributed to the higher disease risk in this age group and partly due to the less established safety and efficacy of lenvatinib in relatively young individuals. The number of AEs in Japan and the United States is significantly higher than that in other countries, potentially due to a larger user base for the drug. This underlying trend may be attributed to factors such as larger population size, stronger reporting willingness, earlier market entry, and earlier expansion of indications, which collectively have facilitated its broader usage.

In our study, the most common and significant SOC level AEs, such as gastrointestinal disorders, nervous system disorders, metabolism and nutrition disorders, respiratory, thoracic and mediastinal disorders, vascular disorders, renal and urinary disorders, hepatobiliary disorders, and endocrine disorders, were consistent with the safety data from labeling and clinical trials. Among the PTs corresponding to gastrointestinal disorders, the top three reported PTs were diarrhea, vomiting, and stomatitis. Multiple clinical trials have also identified diarrhea (incidence rate of 45%–67%) as the most common gastrointestinal event associated with lenvatinib, with nausea, vomiting, and decreased appetite reported by 18–75% of patients [13], and the incidence rate of stomatitis reached 29–32% [14,15]. In terms of nervous system disorders at the PT level, hepatic encephalopathy had the highest number of reports. In a study by Chen involving 240 patients with hepatocellular carcinoma (HCC) treated with lenvatinib, the incidence rate of hepatic encephalopathy was 11.3%. It was also found that the incidence of hepatic encephalopathy in off-label patients with Child-Pugh score>7 was nearly triple that in on-label patients (Child-Pugh A) [16]. Decreased appetite was frequently observed in patients corresponding to metabolism and nutrition disorders. One study indicated that 38.9% of patients with unresectable hepatocellular carcinoma experienced decreased appetite following lenvatinib treatment [17]. Combined with the complex and frequent gastrointestinal adverse reactions such as diarrhea and vomiting, a series of metabolism and nutrition disorders including dehydration, decreased weight, and electrolyte imbalances occurred. PT-level AEs from the other common SOC-level, such as dysphonia, hypertension, hemorrhagic events, proteinuria, hypothyroidism, hyperthyroidism, adrenal insufficiency, and thyroiditis, can occur in more than 20% of patients [18], indicating the multi-system toxicity of lenvatinib affecting the liver, kidney, cardiovascular system, and endocrine system. Furthermore, our study found that fistula is an AE involving multiple systems including respiratory, digestive, reproductive, and vascular systems. In line with clinical investigations, our study yielded results that support the findings of previous research conducted on patients with radioiodine-refractory differentiated thyroid cancer (RAI-R DTC). Specifically, the clinical trial revealed that among RAI-R DTC patients with locally advanced or metastatic conditions who received lenvatinib treatment, approximately 14.7% experienced the development of a fistula or organ perforation. In addition, assessment of risk factors associated with the occurrence of a fistula or organ perforation demonstrated a significant correlation between the presence of tumor infiltration and tumor histology [19].

In our analysis, we have also identified additional significant AE signals that have not been previously reported in regulated trials. These novel AE signals include limbic encephalitis, hypopituitarism, hypophysitis, failure to thrive, pulmonary cavitation, polycythemia, interstitial lung disease, pneumothorax, and spontaneous pneumothorax. These adverse reactions, which may manifest as a consequence of prolonged drug exposure, have also been documented in certain clinical studies or individual case reports. Endocrine-related toxicities are not uncommon in tyrosine kinase inhibitor (TKI) drug therapy. TKI drugs can impact multiple hormone levels by interfering with several endocrine gland axes, leading to disrupted pituitary function and potential deficiencies in growth hormone and insulin-like growth factors, ultimately resulting in failure to thrive [20,21]. Research has found that pulmonary cavitation and pneumothorax primarily occur in lung metastasis patients with tumors, such as in patients with pulmonary metastasis from Anaplastic thyroid cancer treated with lenvatinib, where 46.2% of patients experienced pulmonary cavitation. This is believed to be the result of rapid shrinkage of pulmonary metastases [22]. The mechanism underlying polycythemia may be related to increased erythropoietin (EPO) production. Lenvatinib, as a type of TKI and one of the most potent inhibitors of VEGFR, can synergistically promote the production of EPO in hepatocellular carcinoma cells under hypoxic conditions, leading to the occurrence of polycythemia [23]. Interstitial lung disease may be drug-induced pneumonia caused by the direct toxicity of medications or allergic reactions. However, research has also found that the inhibition of VEGFR may induce apoptosis of alveolar epithelial cells, potentially contributing to the development of interstitial lung disease [8]. Other adverse reactions not yet reported, such as limbic encephalitis, may also be caused by the direct toxicity of drugs or allergic reactions. However, the specific mechanisms are still awaiting further exploration. These findings suggest that closer monitoring of endocrine function, pulmonary imaging examinations, and hematologic indicators is necessary for patients receiving lenvatinib, with timely symptomatic interventions if needed. And these discoveries underscore the critical importance of continuous monitoring for drug-related adverse reactions and serves as a valuable reference for informed decision-making regarding drug selection.

Despite the documentation of AEs including rash, dizziness, cough, insomnia, dysgeusia, alopecia, and hepatotoxicity in certain clinical trials and guidelines, our comprehensive data analysis did not indicate any noteworthy signals for these specific AEs. We propose that this discrepancy may be attributed to reporting biases among different reporting entities, including patients, physicians, and pharmaceutical companies. In clinical trials, adverse reactions are recorded with uniform and strict standards, while reporting in the FAERS database is voluntary. Adverse reactions that have minimal impact may be overlooked during the reporting process, leading to underestimation in the data. However, we cannot disregard the advantages of the large-scale and real-world nature of the FAERS database. These compelling findings may consequently furnish substantive evidence to inform future revisions for lenvatinib's prescribing information.

Currently, there is a paucity of comprehensive real-world large-sample safety studies on lenvatinib based on the FAERS database, the studies retrieved in PubMed mostly focused on a single population or a single adverse reaction [[24], [25], [26], [27]]. Other safety research surrounding lenvatinib primarily consists of meta-analyses or short-term clinical trials, limiting the scope and reliability of available data regarding sample sizes, follow-up durations, and the comprehensive assessment of observed AEs. Additionally, crucial details regarding the precise onset timing of these AEs remain indeterminate. However, with great excitement, our study encompasses the most extensive aggregate of lenvatinib-related cases to date, encompassing an impressive compilation of 89,228 reported cases and 254,886 AEs. Beyond consolidating previously cataloged adverse reactions aligned with the drug's label and established clinical trials, our findings have unearthed a multitude of novel and unanticipated significant AEs. Furthermore, an analysis of the occurrence timing and severe outcomes of AEs, including severity and proportions, has been conducted. All these findings provided comprehensive and invaluable insights into the safety profile of lenvatinib.

This study capitalizes on the inherent advantages of a real-world large-sample investigation coupled with sophisticated data mining techniques. Nevertheless, it is essential to acknowledge and address several limitations that necessitate careful consideration. 1) The FAERS database constitutes a spontaneously reported system, potentially leading to incomplete and inaccurate information collected from diverse countries and healthcare professionals, and thereby introducing analysis biases. For example, rare AEs may be overemphasized in reporting by healthcare professionals, while AEs with minimal impact on patients may be underreported. 2) Very rare adverse events related to lenvatinib use may not exhibit statistical significance in disproportionality calculations due to low occurrence. There may be additional safety signals yet to be identified. 3) The demographics observed in the FAERS reports may not accurately reflect real-world clinical usage and outcomes with lenvatinib due to potential selection bias in reporting. For example, in different stages of various cancer types, the utilization of Lenvatinib in first-line or subsequent-line treatments varies, while advanced-stage patients often present with poorer physical indicators, thus introducing bias into the outcomes. 4) The specificity in attributing AEs to lenvatinib is constrained, with potential influence from concomitant medications (such as ICIs). 5) The effects of changes in lenvatinib dosage, renal impairment, hepatic dysfunction, and other intrinsic/extrinsic factors over time could not be fully accounted for. 6) Due to the absence of a total number encompassing the total population receiving lenvatinib treatment, the calculation of precise incidence rates for each AE remains unfeasible. 7) It is important to note that the utilization of disproportionality analysis solely enables the determination of statistical significance predicated on signal strength, hence lacking definitive evidence regarding causality.

In light of these aforementioned limitations, it is imperative to underscore the necessity of further investigational endeavors aimed at validating these results. Notwithstanding these limitations, the present findings serve as pivotal guidance for healthcare professionals, facilitating closer patient monitoring and diligent surveillance of lenvatinib-associated adverse reactions within the clinical setting.

## 5 Conclusions

Our study entailed a meticulous analysis of real-world data extracted from the FAERS database, a comprehensive endeavor aimed at unearthing invaluable insights into the safety profile and latent risks associated with lenvatinib administration. Through rigorous examination, we empirically observed an array of unforeseen and atypical ADRs, including hypopituitarism and hypophysitis, etc. However, it remains imperative to engage in further prospective clinical investigations to corroborate and amplify our comprehension of the intricate relationship between lenvatinib utilization and the manifestation of these distinctive ADRs. Leveraging an innovative and distinctive paradigm, this research endeavor proffers a pioneering methodology to probe the implications of AEs germane to medication utilization.

## Data availability statement

The public datasets to support the results of this subject can be gained from the Food and Drug Administration's Adverse Event Reporting System (FAERS) public database: https://fis.fda.gov/extensions/FPD-QDE-FAERS/FPD-QDE-FAERS.html.

## Ethics statement

Not applicable.

## Funding

This work was funded by the 10.13039/501100001809National Natural Science Foundation of China (82170541).

## CRediT authorship contribution statement

**Zhe Yu:** Conceptualization, Writing – original draft, Writing – review & editing. **Jing Luo:** Data curation, Formal analysis, Methodology, Software. **Hongshan Wei:** Funding acquisition, Project administration, Writing – review & editing.

## Declaration of competing interest

The authors declare that they have no known competing financial interests or personal relationships that could have appeared to influence the work reported in this paper.
