# Supplementary material for: Novel insights into post-marketing adverse events associated with lenvatinib: A comprehensive analysis utilizing the FAERS database
Source: Heliyon. 2024 Mar 13;10(6):e28132. doi: 10.1016/j.heliyon.2024.e28132 (PMC10958715; doi:10.1016/j.heliyon.2024.e28132)
Supplement: Multimedia component 1 [file mmc1.docx]

| **Supplementary Table S1** Standardization of the drug name for Lenvatinib | | |
| --- | --- | --- |
| **Pharmaceutical trade name** | **Active Ingredient** | **Standard Name** |
| Lenvatinib | Lenvatinib | Lenvatinib |
| Lodatir | Lenvatinib | Lenvatinib |
| Lenvatinib Mesilate | Lenvatinib Mesilate | Lenvatinib |
| Lenvatinib Mesylate | Lenvatinib Mesilate | Lenvatinib |
| Lenvima | Lenvatinib Mesilate | Lenvatinib |
| Kisplyx | Lenvatinib Mesilate | Lenvatinib |
| Lenvatib | Lenvatinib Mesilate | Lenvatinib |
| Lenvixi | Lenvatinib Mesilate | Lenvatinib |
| Bdfoie | Lenvatinib Mesilate | Lenvatinib |
| Glenvas | Lenvatinib Mesilate | Lenvatinib |
| Lentib | Lenvatinib Mesilate | Lenvatinib |
| Lenvatol | Lenvatinib Mesilate | Lenvatinib |
| Lentykine | Lenvatinib Mesilate | Lenvatinib |
| Lentris | Lenvatinib Mesilate | Lenvatinib |
| Lenvenib | Lenvatinib Mesilate | Lenvatinib |
| Lenced | Lenvatinib Mesilate | Lenvatinib |
| Lenvat | Lenvatinib Mesilate | Lenvatinib |
| Adlante | Lenvatinib Mesilate | Lenvatinib |
| Lenvonco | Lenvatinib Mesilate | Lenvatinib |
| Lenvatinib Eczane | Lenvatinib Mesilate | Lenvatinib |
