# Supplementary material for: Novel insights into post-marketing adverse events associated with lenvatinib: A comprehensive analysis utilizing the FAERS database
Source: Heliyon. 2024 Mar 13;10(6):e28132. doi: 10.1016/j.heliyon.2024.e28132 (PMC10958715; doi:10.1016/j.heliyon.2024.e28132)
Supplement: Multimedia component 2 [file mmc2.docx]

**Supplementary Table S2** Quadruple table of AEs

|  | Lenvatinib | Non-Lenvatinib |
| --- | --- | --- |
| Target AEs | a | c |
| Non-target AEs | b | d |
