# Supplementary material for: Novel insights into post-marketing adverse events associated with lenvatinib: A comprehensive analysis utilizing the FAERS database
Source: Heliyon. 2024 Mar 13;10(6):e28132. doi: 10.1016/j.heliyon.2024.e28132 (PMC10958715; doi:10.1016/j.heliyon.2024.e28132)
Supplement: Multimedia component 3 [file mmc3.docx]

**Supplementary Table S3 Four major algorithms used for signal detection.**

| Algorithms | Equation | Criteria |
| --- | --- | --- |
| ROR | $ROR=\frac{(a/c)}{(b/d)}=\frac{\mathrm{ad}}{\mathrm{bc}}$ | a≥3, lower limit of 95% CI>1 |
|  | $95\%CI=e^{ln(ROR)\pm1.96\sqrt{(\frac{1}{a}+\frac{1}{b}+\frac{1}{c}+\frac{1}{d})}}$ |  |
| PRR | $PRR＝\frac{a/(a+b)}{c/(c+d)}$ | a≥3, PRR≥2, χ2≥4 |
|  | $\chi2 =\frac{{(ad-bc)}^{2}(a+b+c+d)}{( a+b)(a+c)(c+d)(b+d)}$ |  |
| BCPNN | IC=$\log_{2}\frac{a(a+b+c+d)}{(a+b)(a+c)}$ | a≥3, IC025>0 |
|  | E(IC)=$\log_{2}\frac{(a+\gamma11)(a+b+c+d+\alpha)(a+b+c+d+\beta)}{（a+b+c+d+\gamma）(a+b+\alpha1)(a+c+\beta1)}$ |  |
|  | V(IC)=$\frac{1}{{(ln2)}^{2}}\{\left[ \frac{\left( a+b+c+d \right)-a+\gamma-\gamma11}{\left( a+\gamma11 \right)\left( 1+a+b+c+d+\gamma\right)} \right]+\left[ \frac{\left( a+b+c+d \right)-\left( a+b \right)+\alpha-\alpha1}{\left( a+b+\alpha1 \right)\left( 1+a+b+c+d+\alpha\right)} \right]+\left[ \frac{\left( a+b+c+d \right)-\left( a+c \right)+\beta-\beta1}{\left( a+c+\beta1 \right)\left( 1+a+b+c+d+\beta\right)} \right]\}$ |  |
|  | $\gamma=\gamma11\frac{(a+b+c+d+\alpha)(a+b+c+d+\beta)}{(a+b+\alpha1)(a+c+\beta1)}$ |  |
|  | 95%CI=E(IC)±2$\sqrt{V(IC)}$ |  |
|  | $\alpha1=\beta1=1；\alpha=\beta=2；\gamma11=1$ |  |
| MGPS | $EBGM＝\frac{a(a+b+c+d)}{(a+c)(a+b)}$ | EBGM05>2 |
|  | $95\%CI=e^{ln(EBGM)\pm1.96\sqrt{(\frac{1}{a}+\frac{1}{b}+\frac{1}{c}+\frac{1}{d})}}$ |  |

Equation: a, number of reports containing both the target drug and target adverse drug reaction; b, number of reports containing other adverse drug reaction of the target drug; c, number of reports containing the target adverse drug reaction of other drugs; d, number of reports containing other drugs and other adverse drug reactions. 95%CI, 95% confidence interval; χ^2^, chi-squared; IC, information component; E(IC), the IC expectations; EBGM, empirical Bayesian geometric mean; EBGM05, the lower limit of 95% CI of EBGM.
