# Supplementary material for: Novel insights into post-marketing adverse events associated with lenvatinib: A comprehensive analysis utilizing the FAERS database
Source: Heliyon. 2024 Mar 13;10(6):e28132. doi: 10.1016/j.heliyon.2024.e28132 (PMC10958715; doi:10.1016/j.heliyon.2024.e28132)
Supplement: Multimedia component 4 [file mmc4.docx]

**Supplementary Table S4** Signal strength of reports of Lenvatinib at the Preferred Term (PT) level in the FAERS database.

| **SOC** | **Preferred terms (PTs)** | **Cases Numbers** | **ROR (95% CI)** | **PRR (χ2)** | **IC (95% CI)** | **EBGM (95% CI)** |
| --- | --- | --- | --- | --- | --- | --- |
| Blood and lymphatic system disorders | Disseminated intravascular coagulation | 28 | 3.19(2.20-4.63) | 3.19(41.94) | 1.67(1.03-2.1) | 3.18(2.19-4.61) |
|  | Polycythaemia* | 11 | 8.25(4.55-14.95) | 8.24(69.16) | 3.03(1.51-3.19) | 8.15(4.5-14.78) |
| Cardiac disorders | Cardiac failure | 199 | 3.18(2.77-3.66) | 3.18(295.64) | 1.66(1.44-1.85) | 3.17(2.75-3.64) |
|  | Acute myocardial infarction | 59 | 2.77(2.15-3.58) | 2.77(66.41) | 1.47(1.05-1.8) | 2.76(2.14-3.57) |
|  | Myocarditis | 48 | 5.64(4.24-7.49) | 5.64(181.48) | 2.48(1.94-2.77) | 5.6(4.21-7.44) |
|  | Acute coronary syndrome | 24 | 4.05(2.71-6.04) | 4.04(54.68) | 2.01(1.27-2.42) | 4.03(2.69-6.01) |
|  | Cardiac dysfunction | 19 | 5.89(3.75-9.25) | 5.89(76.37) | 2.55(1.58-2.88) | 5.84(3.72-9.18) |
|  | Immune-mediated myocarditis | 6 | 9.43(4.21-21.11) | 9.43(44.55) | 3.22(0.99-3.19) | 9.31(4.16-20.84) |
| Congenital, familial and genetic disorders | Tracheo-oesophageal fistula | 21 | 46.23(29.70-71.97) | 46.21(867.58) | 5.43(3.25-4.52) | 43.23(27.77-67.3) |
| Endocrine disorders | Hypothyroidism | 385 | 17.39(15.71-19.25) | 17.27(5751.59) | 4.07(3.87-4.17) | 16.85(15.22-18.65) |
|  | Adrenal insufficiency | 103 | 11.89(9.78-14.45) | 11.87(1007.03) | 3.55(3.12-3.69) | 11.67(9.61-14.19) |
|  | Hyperthyroidism | 71 | 6.25(4.94-7.89) | 6.24(309.39) | 2.63(2.19-2.87) | 6.19(4.9-7.82) |
|  | Thyroiditis | 41 | 17.45(12.79-23.80) | 17.43(618.69) | 4.09(3.17-4.07) | 17.01(12.47-23.19) |
|  | Thyroid disorder | 39 | 3.18(2.32-4.35) | 3.18(57.86) | 1.66(1.13-2.04) | 3.16(2.31-4.34) |
|  | Hypophysitis* | 15 | 7.18(4.32-11.94) | 7.18(78.88) | 2.83(1.64-3.09) | 7.11(4.27-11.83) |
|  | Primary adrenal insufficiency | 9 | 54.47(27.60-107.53) | 54.46(436.02) | 5.65(2.13-4.03) | 50.35(25.51-99.4) |
|  | Hypopituitarism* | 9 | 5.98(3.10-11.53) | 5.98(36.97) | 2.57(1.07-2.91) | 5.93(3.08-11.44) |
|  | Adrenocorticotropic hormone deficiency | 8 | 10.99(5.46-22.10) | 10.98(71.41) | 3.44(1.4-3.34) | 10.82(5.38-21.76) |
|  | Adrenocortical insufficiency acute | 6 | 4.50(2.02-10.05) | 4.50(16.23) | 2.16(0.49-2.68) | 4.48(2.01-10) |
|  | Silent thyroiditis | 3 | 23.91(7.56-75.68) | 23.91(63.54) | 4.53(0.35-3.3) | 23.1(7.3-73.12) |
|  | Adrenal haemorrhage | 3 | 7.05(2.26-22.00) | 7.05(15.42) | 2.8(0.03-2.94) | 6.99(2.24-21.8) |
| Eye disorders | Retinal vascular occlusion | 4 | 5.62(2.10-15.04) | 5.62(15.07) | 2.48(0.24-2.84) | 5.58(2.09-14.94) |
| Gastrointestinal disorders | Diarrhoea | 1917 | 3.60(3.44-3.77) | 3.50(3447.94) | 1.8(1.73-1.87) | 3.49(3.33-3.65) |
|  | Vomiting | 1022 | 2.91(2.74-3.10) | 2.87(1252.19) | 1.52(1.42-1.61) | 2.87(2.69-3.05) |
|  | Stomatitis | 440 | 8.76(7.97-9.63) | 8.70(2960.35) | 3.1(2.94-3.22) | 8.59(7.82-9.45) |
|  | Abdominal pain | 419 | 2.36(2.14-2.60) | 2.35(324.47) | 1.23(1.08-1.37) | 2.34(2.13-2.58) |
|  | Constipation | 389 | 2.24(2.03-2.48) | 2.23(264.97) | 1.16(1.01-1.3) | 2.23(2.02-2.46) |
|  | Ascites | 216 | 9.64(8.42-11.02) | 9.60(1640.41) | 3.24(2.99-3.39) | 9.47(8.28-10.84) |
|  | Oral pain | 192 | 10.22(8.86-11.79) | 10.19(1566.99) | 3.33(3.05-3.47) | 10.05(8.71-11.59) |
|  | Dry mouth | 188 | 3.07(2.66-3.55) | 3.06(260.62) | 1.61(1.38-1.81) | 3.06(2.65-3.53) |
|  | Pancreatitis | 172 | 5.16(4.44-5.99) | 5.14(569.94) | 2.35(2.1-2.54) | 5.11(4.4-5.94) |
|  | Colitis | 134 | 4.49(3.79-5.32) | 4.48(360.15) | 2.16(1.87-2.37) | 4.46(3.76-5.28) |
|  | Haematemesis | 97 | 5.15(4.22-6.29) | 5.14(321.32) | 2.35(2-2.59) | 5.11(4.18-6.24) |
|  | Intestinal perforation | 95 | 11.44(9.33-14.01) | 11.42(887.46) | 3.49(3.05-3.64) | 11.24(9.17-13.77) |
|  | Glossodynia | 83 | 5.39(4.34-6.69) | 5.38(293.98) | 2.42(2.03-2.66) | 5.35(4.31-6.64) |
|  | Oesophageal varices haemorrhage | 79 | 47.27(37.61-59.40) | 47.20(3331.72) | 5.46(4.51-5.17) | 44.09(35.08-55.4) |
|  | Gastrointestinal perforation | 77 | 24.02(19.13-30.16) | 23.99(1636.30) | 4.53(3.84-4.51) | 23.17(18.46-29.1) |
|  | Large intestine perforation | 54 | 10.51(8.03-13.75) | 10.50(456.73) | 3.37(2.75-3.54) | 10.35(7.91-13.54) |
|  | Pancreatitis acute | 53 | 3.46(2.64-4.53) | 3.45(92.00) | 1.78(1.33-2.11) | 3.44(2.63-4.51) |
|  | Upper gastrointestinal haemorrhage | 53 | 3.30(2.52-4.33) | 3.30(84.53) | 1.72(1.26-2.05) | 3.29(2.51-4.31) |
|  | Oral discomfort | 47 | 4.21(3.16-5.61) | 4.21(114.21) | 2.07(1.55-2.39) | 4.19(3.14-5.58) |
|  | Gastric haemorrhage | 41 | 4.45(3.27-6.05) | 4.45(108.82) | 2.15(1.58-2.48) | 4.42(3.25-6.01) |
|  | Ileus | 36 | 4.33(3.12-6.01) | 4.33(91.55) | 2.11(1.51-2.46) | 4.31(3.1-5.98) |
|  | Enterocolitis | 35 | 7.81(5.60-10.91) | 7.81(205.39) | 2.95(2.22-3.19) | 7.73(5.54-10.79) |
|  | Tongue discomfort | 30 | 6.41(4.47-9.18) | 6.41(135.53) | 2.67(1.92-2.96) | 6.35(4.43-9.1) |
|  | Mouth haemorrhage | 28 | 5.03(3.47-7.30) | 5.03(89.77) | 2.32(1.6-2.67) | 5(3.45-7.25) |
|  | Small intestinal perforation | 26 | 19.56(13.24-28.90) | 19.55(444.47) | 4.25(2.95-4.07) | 19.02(12.87-28.09) |
|  | Gastric perforation | 25 | 11.91(8.02-17.70) | 11.91(245.37) | 3.55(2.48-3.62) | 11.71(7.89-17.4) |
|  | Oesophageal fistula | 24 | 115.39(74.76-178.10) | 115.34(2312.27) | 6.62(3.71-4.95) | 98.19(63.61-151.55) |
|  | Odynophagia | 23 | 5.58(3.70-8.41) | 5.58(85.69) | 2.47(1.63-2.81) | 5.54(3.67-8.35) |
|  | Gingival pain | 23 | 3.93(2.61-5.92) | 3.92(49.84) | 1.97(1.21-2.39) | 3.91(2.59-5.89) |
|  | Intra-abdominal fluid collection | 22 | 10.45(6.85-15.92) | 10.44(184.89) | 3.36(2.27-3.48) | 10.29(6.75-15.69) |
|  | Faeces soft | 22 | 3.35(2.21-5.10) | 3.35(36.14) | 1.74(1-2.2) | 3.34(2.2-5.08) |
|  | Oral mucosal blistering | 21 | 3.53(2.30-5.42) | 3.53(37.85) | 1.81(1.04-2.27) | 3.51(2.29-5.4) |
|  | Duodenal perforation | 18 | 25.70(16.04-41.16) | 25.69(410.91) | 4.63(2.78-4.13) | 24.75(15.45-39.65) |
|  | Duodenal ulcer | 18 | 3.70(2.33-5.89) | 3.70(35.30) | 1.88(1.03-2.36) | 3.69(2.32-5.86) |
|  | Oesophageal perforation | 17 | 25.85(15.92-41.97) | 25.84(390.49) | 4.64(2.72-4.11) | 24.89(15.33-40.42) |
|  | Immune-mediated enterocolitis | 17 | 8.84(5.48-14.27) | 8.84(116.62) | 3.13(1.93-3.3) | 8.73(5.41-14.1) |
|  | Duodenal ulcer haemorrhage | 16 | 6.97(4.26-11.41) | 6.97(80.98) | 2.79(1.65-3.06) | 6.91(4.22-11.31) |
|  | Glossitis | 15 | 5.89(3.54-9.79) | 5.88(60.28) | 2.55(1.44-2.89) | 5.84(3.51-9.71) |
|  | Oesophageal haemorrhage | 14 | 13.42(7.90-22.78) | 13.42(157.64) | 3.72(2.11-3.61) | 13.17(7.76-22.35) |
|  | Intra-abdominal haemorrhage | 14 | 7.94(4.69-13.46) | 7.94(83.94) | 2.97(1.68-3.18) | 7.86(4.64-13.31) |
|  | Pneumatosis intestinalis | 13 | 5.51(3.19-9.51) | 5.51(47.59) | 2.45(1.28-2.83) | 5.47(3.17-9.45) |
|  | Diverticular perforation | 12 | 7.74(4.38-13.68) | 7.74(69.63) | 2.94(1.53-3.15) | 7.66(4.34-13.54) |
|  | Varices oesophageal | 12 | 5.39(3.05-9.52) | 5.39(42.56) | 2.42(1.2-2.81) | 5.35(3.03-9.45) |
|  | Gastric ulcer perforation | 9 | 9.79(5.07-18.91) | 9.79(69.96) | 3.27(1.45-3.29) | 9.66(5-18.65) |
|  | Gastric varices haemorrhage | 7 | 23.83(11.21-50.67) | 23.83(147.71) | 4.53(1.57-3.66) | 23.03(10.83-48.95) |
|  | Enterocutaneous fistula | 7 | 10.74(5.09-22.67) | 10.74(60.83) | 3.4(1.24-3.3) | 10.58(5.01-22.33) |
|  | Tongue erythema | 7 | 8.07(3.83-17.01) | 8.07(42.82) | 3(1.06-3.12) | 7.98(3.79-16.82) |
|  | Rectal ulcer | 7 | 7.78(3.69-16.40) | 7.78(40.88) | 2.95(1.04-3.09) | 7.7(3.65-16.23) |
|  | Rectal perforation | 6 | 16.48(7.33-37.05) | 16.48(85.08) | 4.01(1.24-3.45) | 16.1(7.16-36.19) |
|  | Haemorrhagic ascites | 5 | 26.79(10.95-65.52) | 26.79(119.23) | 4.69(1.12-3.53) | 25.77(10.54-63.03) |
|  | Fistula of small intestine | 5 | 11.93(4.92-28.89) | 11.93(49.16) | 3.55(0.88-3.26) | 11.73(4.84-28.41) |
|  | Enterovesical fistula | 5 | 9.36(3.87-22.64) | 9.36(36.82) | 3.21(0.77-3.15) | 9.25(3.82-22.35) |
|  | Gastrointestinal fistula | 5 | 6.13(2.54-14.79) | 6.13(21.27) | 2.6(0.53-2.9) | 6.08(2.52-14.68) |
|  | Lower gastrointestinal perforation | 4 | 62.25(22.32-173.61) | 62.25(220.09) | 5.83(0.86-3.58) | 56.92(20.41-158.74) |
|  | Plicated tongue | 4 | 10.17(3.79-27.31) | 10.17(32.58) | 3.33(0.53-3.14) | 10.03(3.74-26.93) |
|  | Mechanical ileus | 4 | 8.54(3.19-22.91) | 8.54(26.30) | 3.08(0.46-3.06) | 8.45(3.15-22.65) |
|  | Mesenteric vein thrombosis | 4 | 5.86(2.19-15.69) | 5.86(15.99) | 2.54(0.27-2.86) | 5.82(2.17-15.57) |
|  | Immune-mediated pancreatitis | 3 | 14.31(4.56-44.93) | 14.31(36.35) | 3.81(0.26-3.18) | 14.03(4.47-44.03) |
|  | Mesenteric artery thrombosis | 3 | 10.77(3.44-33.72) | 10.77(26.17) | 3.41(0.18-3.1) | 10.61(3.39-33.22) |
|  | Colonic fistula | 3 | 6.65(2.13-20.73) | 6.65(14.25) | 2.72(0-2.91) | 6.59(2.11-20.55) |
| General disorders and administration site conditions | Fatigue | 1590 | 2.41(2.29-2.53) | 2.37(1265.31) | 1.24(1.16-1.31) | 2.36(2.25-2.48) |
|  | Asthenia | 851 | 2.87(2.68-3.07) | 2.84(1017.29) | 1.5(1.4-1.6) | 2.83(2.65-3.03) |
|  | Mucosal inflammation | 80 | 3.99(3.20-4.97) | 3.98(177.61) | 1.99(1.61-2.26) | 3.96(3.18-4.94) |
|  | Impaired healing | 79 | 3.45(2.77-4.31) | 3.45(136.66) | 1.78(1.41-2.06) | 3.44(2.75-4.29) |
|  | Decreased activity | 33 | 3.64(2.59-5.13) | 3.64(62.82) | 1.86(1.25-2.25) | 3.62(2.57-5.1) |
|  | Non-cardiac chest pain | 10 | 4.63(2.49-8.63) | 4.63(28.28) | 2.2(0.92-2.67) | 4.61(2.47-8.58) |
|  | Early satiety | 8 | 8.18(4.07-16.44) | 8.18(49.82) | 3.02(1.21-3.15) | 8.09(4.03-16.26) |
|  | Perforation | 7 | 8.57(4.06-18.06) | 8.57(46.18) | 3.08(1.1-3.16) | 8.47(4.02-17.85) |
|  | Pneumatosis | 3 | 9.71(3.10-30.35) | 9.71(23.09) | 3.26(0.15-3.06) | 9.58(3.06-29.95) |
| Hepatobiliary disorders | Hepatic function abnormal | 206 | 7.96(6.94-9.13) | 7.93(1233.54) | 2.97(2.72-3.13) | 7.85(6.84-9.01) |
|  | Hepatic failure | 167 | 8.13(6.98-9.47) | 8.10(1027.60) | 3(2.72-3.17) | 8.02(6.88-9.34) |
|  | Cholecystitis | 158 | 22.02(18.79-25.81) | 21.95(3057.49) | 4.41(4-4.47) | 21.27(18.15-24.93) |
|  | Liver disorder | 102 | 3.19(2.63-3.88) | 3.19(152.42) | 1.67(1.35-1.92) | 3.18(2.61-3.86) |
|  | Cholangitis | 80 | 17.91(14.34-22.37) | 17.88(1241.17) | 4.12(3.53-4.18) | 17.43(13.96-21.77) |
|  | Jaundice | 77 | 4.36(3.48-5.45) | 4.35(197.60) | 2.11(1.73-2.38) | 4.33(3.46-5.42) |
|  | Gallbladder disorder | 62 | 6.38(4.97-8.20) | 6.37(278.28) | 2.66(2.18-2.91) | 6.32(4.92-8.12) |
|  | Cholecystitis acute | 56 | 16.20(12.43-21.13) | 16.19(778.74) | 3.98(3.26-4.04) | 15.82(12.13-20.63) |
|  | Hepatitis | 51 | 2.88(2.19-3.79) | 2.88(62.26) | 1.52(1.07-1.87) | 2.87(2.18-3.78) |
|  | Portal vein thrombosis | 42 | 18.70(13.76-25.42) | 18.69(683.56) | 4.19(3.25-4.15) | 18.19(13.39-24.73) |
|  | Immune-mediated hepatitis | 28 | 22.58(15.49-32.91) | 22.56(557.82) | 4.45(3.12-4.21) | 21.85(14.99-31.84) |
|  | Jaundice cholestatic | 17 | 7.15(4.43-11.54) | 7.15(88.96) | 2.82(1.72-3.09) | 7.08(4.39-11.42) |
|  | Hepatic pain | 17 | 4.82(2.99-7.76) | 4.82(51.03) | 2.26(1.3-2.67) | 4.79(2.97-7.72) |
|  | Cholangitis acute | 15 | 24.76(14.79-41.47) | 24.76(329.48) | 4.58(2.56-4.03) | 23.89(14.27-40.01) |
|  | Hepatic haemorrhage | 14 | 25.57(14.99-43.61) | 25.56(317.94) | 4.62(2.5-4.02) | 24.63(14.44-42.02) |
|  | Haemobilia | 13 | 41.66(23.78-72.99) | 41.65(484.86) | 5.29(2.6-4.19) | 39.21(22.38-68.7) |
|  | Gallbladder rupture | 12 | 20.22(11.38-35.91) | 20.21(212.59) | 4.3(2.2-3.82) | 19.64(11.05-34.88) |
|  | Biliary obstruction | 12 | 5.20(2.95-9.17) | 5.20(40.36) | 2.37(1.16-2.77) | 5.16(2.93-9.11) |
|  | Biliary colic | 10 | 4.80(2.58-8.94) | 4.80(29.85) | 2.25(0.95-2.7) | 4.77(2.56-8.89) |
|  | Hepatorenal syndrome | 9 | 7.22(3.74-13.92) | 7.22(47.68) | 2.84(1.23-3.06) | 7.15(3.71-13.79) |
|  | Portal vein occlusion | 5 | 54.47(21.87-135.64) | 54.46(242.23) | 5.65(1.22-3.68) | 50.35(20.22-125.39) |
|  | Gallbladder enlargement | 5 | 6.37(2.64-15.37) | 6.37(22.42) | 2.66(0.56-2.93) | 6.32(2.62-15.25) |
|  | Ischaemic hepatitis | 5 | 5.88(2.44-14.18) | 5.88(20.06) | 2.54(0.51-2.88) | 5.83(2.42-14.07) |
|  | Biliary fistula | 4 | 37.35(13.64-102.31) | 37.35(133.85) | 5.14(0.83-3.5) | 35.38(12.92-96.92) |
|  | Hepatic vein thrombosis | 4 | 17.20(6.37-46.42) | 17.20(59.47) | 4.07(0.7-3.32) | 16.78(6.22-45.3) |
|  | Hepatic infarction | 3 | 11.14(3.56-34.88) | 11.14(27.23) | 3.46(0.19-3.11) | 10.97(3.5-34.35) |
|  | Hepatorenal failure | 3 | 7.08(2.27-22.08) | 7.08(15.49) | 2.81(0.03-2.94) | 7.01(2.25-21.88) |
| Infections and infestations | Pneumonia aspiration | 65 | 3.39(2.65-4.32) | 3.38(108.63) | 1.75(1.35-2.06) | 3.37(2.64-4.3) |
|  | Gastroenteritis | 32 | 2.98(2.11-4.22) | 2.98(41.90) | 1.57(0.98-1.99) | 2.97(2.1-4.2) |
|  | Cholecystitis infective | 23 | 10.92(7.23-16.49) | 10.92(203.80) | 3.43(2.34-3.53) | 10.75(7.12-16.24) |
|  | Liver abscess | 22 | 8.73(5.73-13.30) | 8.73(148.61) | 3.11(2.09-3.3) | 8.63(5.67-13.14) |
|  | Anal abscess | 20 | 4.41(2.84-6.84) | 4.40(52.28) | 2.13(1.28-2.55) | 4.38(2.82-6.8) |
|  | Gingivitis | 16 | 3.91(2.39-6.40) | 3.91(34.50) | 1.96(1.03-2.44) | 3.9(2.38-6.37) |
|  | Lung abscess | 12 | 7.56(4.28-13.35) | 7.56(67.48) | 2.9(1.51-3.12) | 7.48(4.23-13.22) |
|  | Infectious pleural effusion | 10 | 7.54(4.04-14.06) | 7.54(56.07) | 2.9(1.36-3.11) | 7.46(4-13.92) |
|  | Necrotising fasciitis | 10 | 3.87(2.08-7.20) | 3.87(21.14) | 1.95(0.74-2.48) | 3.85(2.07-7.17) |
|  | Perineal abscess | 4 | 9.68(3.61-25.99) | 9.68(30.69) | 3.26(0.51-3.12) | 9.56(3.56-25.65) |
|  | Abscess neck | 4 | 7.07(2.64-18.93) | 7.07(20.61) | 2.81(0.37-2.97) | 7(2.61-18.75) |
|  | Enterocolitis infectious | 4 | 6.64(2.48-17.77) | 6.64(18.95) | 2.72(0.34-2.93) | 6.58(2.46-17.62) |
|  | Vulvitis | 3 | 14.31(4.56-44.93) | 14.31(36.35) | 3.81(0.26-3.18) | 14.03(4.47-44.03) |
|  | Urinary tract infection staphylococcal | 3 | 10.83(3.46-33.91) | 10.83(26.34) | 3.42(0.18-3.1) | 10.67(3.41-33.4) |
|  | Abdominal wall abscess | 3 | 6.74(2.16-21.02) | 6.74(14.51) | 2.74(0.01-2.92) | 6.68(2.14-20.83) |
| Injury, poisoning and procedural complications | Tracheal haemorrhage | 22 | 91.62(58.64-143.16) | 91.58(1728.85) | 6.33(3.53-4.81) | 80.45(51.49-125.7) |
|  | Wound dehiscence | 14 | 6.26(3.70-10.60) | 6.26(61.27) | 2.63(1.45-2.95) | 6.21(3.67-10.51) |
|  | Hepatic rupture | 7 | 54.47(25.19-117.78) | 54.46(339.12) | 5.65(1.75-3.87) | 50.35(23.29-108.87) |
|  | Tracheostomy malfunction | 4 | 25.14(9.26-68.25) | 25.14(89.28) | 4.6(0.78-3.42) | 24.24(8.93-65.82) |
|  | Vascular pseudoaneurysm ruptured | 3 | 23.07(7.29-72.96) | 23.07(61.17) | 4.48(0.34-3.29) | 22.32(7.06-70.58) |
|  | Gastroenteritis radiation | 3 | 16.76(5.33-52.72) | 16.76(43.34) | 4.03(0.29-3.22) | 16.36(5.2-51.48) |
| Investigations | Blood pressure increased | 1162 | 9.55(9.01-10.13) | 9.36(8570.24) | 3.21(3.11-3.28) | 9.24(8.71-9.79) |
|  | Weight decreased | 729 | 3.19(2.96-3.43) | 3.16(1073.54) | 1.65(1.54-1.76) | 3.15(2.92-3.38) |
|  | Platelet count decreased | 432 | 5.07(4.61-5.58) | 5.04(1390.37) | 2.32(2.17-2.45) | 5.01(4.55-5.51) |
|  | Ammonia increased | 172 | 46.42(39.76-54.19) | 46.26(7113.78) | 5.44(4.89-5.35) | 43.27(37.06-50.52) |
|  | Blood bilirubin increased | 156 | 8.81(7.52-10.32) | 8.79(1062.54) | 3.12(2.82-3.28) | 8.68(7.41-10.17) |
|  | Aspartate aminotransferase increased | 120 | 3.81(3.19-4.56) | 3.81(246.88) | 1.92(1.63-2.15) | 3.79(3.17-4.53) |
|  | Blood creatinine increased | 116 | 2.46(2.05-2.95) | 2.46(100.09) | 1.29(1.01-1.54) | 2.45(2.04-2.94) |
|  | Alanine aminotransferase increased | 114 | 2.96(2.46-3.56) | 2.95(146.68) | 1.56(1.26-1.8) | 2.94(2.45-3.54) |
|  | Blood potassium decreased | 93 | 4.11(3.35-5.04) | 4.11(217.40) | 2.03(1.69-2.28) | 4.09(3.33-5.01) |
|  | Blood sodium decreased | 86 | 6.69(5.41-8.27) | 6.68(411.08) | 2.73(2.33-2.95) | 6.62(5.35-8.19) |
|  | Blood thyroid stimulating hormone increased | 79 | 9.72(7.79-12.15) | 9.71(608.36) | 3.26(2.79-3.44) | 9.58(7.67-11.97) |
|  | C-reactive protein increased | 74 | 2.68(2.14-3.37) | 2.68(77.80) | 1.42(1.05-1.72) | 2.68(2.13-3.36) |
|  | Blood magnesium decreased | 58 | 8.25(6.37-10.69) | 8.24(364.46) | 3.03(2.48-3.24) | 8.15(6.29-10.56) |
|  | Protein urine present | 56 | 14.50(11.13-18.90) | 14.49(688.08) | 3.83(3.14-3.91) | 14.2(10.89-18.5) |
|  | Thyroid function test abnormal | 41 | 12.69(9.32-17.29) | 12.68(432.82) | 3.64(2.84-3.74) | 12.46(9.15-16.97) |
|  | Blood calcium decreased | 39 | 4.47(3.26-6.13) | 4.47(104.31) | 2.15(1.57-2.49) | 4.45(3.24-6.09) |
|  | Blood potassium increased | 39 | 3.22(2.35-4.42) | 3.22(59.50) | 1.68(1.15-2.06) | 3.21(2.34-4.4) |
|  | Blood albumin decreased | 38 | 8.20(5.96-11.30) | 8.20(237.16) | 3.02(2.31-3.24) | 8.11(5.89-11.17) |
|  | Ejection fraction decreased | 35 | 2.84(2.04-3.95) | 2.84(41.42) | 1.5(0.95-1.91) | 2.83(2.03-3.94) |
|  | Blood pressure diastolic increased | 29 | 7.34(5.09-10.59) | 7.34(157.08) | 2.86(2.06-3.12) | 7.27(5.04-10.48) |
|  | Lipase increased | 28 | 5.47(3.77-7.93) | 5.46(101.29) | 2.44(1.7-2.77) | 5.43(3.74-7.87) |
|  | Amylase increased | 19 | 6.21(3.95-9.76) | 6.21(82.31) | 2.62(1.64-2.94) | 6.16(3.92-9.68) |
|  | Thyroid hormones decreased | 17 | 21.21(13.08-34.38) | 21.20(316.99) | 4.36(2.61-3.99) | 20.57(12.69-33.34) |
|  | Tumour marker increased | 17 | 3.87(2.40-6.23) | 3.87(35.91) | 1.94(1.05-2.41) | 3.85(2.39-6.2) |
|  | Thyroglobulin increased | 14 | 102.84(58.53-180.68) | 102.81(1219.63) | 6.48(2.9-4.49) | 88.97(50.64-156.32) |
|  | Alpha 1 foetoprotein increased | 11 | 20.66(11.34-37.66) | 20.66(199.48) | 4.33(2.11-3.8) | 20.06(11-36.56) |
|  | Blood potassium abnormal | 9 | 4.17(2.17-8.04) | 4.17(21.57) | 2.05(0.74-2.57) | 4.15(2.16-8) |
|  | Thyroglobulin decreased | 7 | 120.41(53.77-269.65) | 120.40(699.90) | 6.67(1.8-4.01) | 101.82(45.47-228.02) |
|  | Blood electrolytes decreased | 7 | 6.41(3.04-13.49) | 6.41(31.64) | 2.67(0.9-2.95) | 6.36(3.02-13.38) |
|  | Thyroid hormones increased | 6 | 5.46(2.45-12.20) | 5.46(21.69) | 2.44(0.64-2.83) | 5.42(2.43-12.12) |
|  | Prothrombin level decreased | 5 | 12.57(5.19-30.46) | 12.57(52.24) | 3.63(0.9-3.28) | 12.35(5.1-29.92) |
|  | Protein induced by vitamin K absence or antagonist II increased | 4 | 93.38(32.75-266.22) | 93.37(319.83) | 6.35(0.87-3.64) | 81.82(28.7-233.28) |
|  | Blood magnesium abnormal | 4 | 8.98(3.35-24.10) | 8.98(28.00) | 3.15(0.48-3.08) | 8.88(3.31-23.81) |
|  | Blood magnesium increased | 4 | 6.27(2.34-16.78) | 6.27(17.55) | 2.64(0.31-2.9) | 6.22(2.32-16.65) |
|  | Protein urine | 4 | 5.61(2.10-15.01) | 5.61(15.02) | 2.48(0.24-2.84) | 5.57(2.08-14.91) |
|  | Blood albumin abnormal | 3 | 9.12(2.92-28.50) | 9.12(21.39) | 3.17(0.13-3.04) | 9.01(2.88-28.15) |
| Metabolism and nutrition disorders | Decreased appetite | 1764 | 9.42(8.98-9.88) | 9.12(12632.42) | 3.17(3.09-3.24) | 9.01(8.59-9.45) |
|  | Dehydration | 912 | 9.52(8.92-10.17) | 9.37(6735.81) | 3.21(3.1-3.29) | 9.25(8.66-9.88) |
|  | Hyponatraemia | 173 | 4.05(3.49-4.71) | 4.04(393.98) | 2.01(1.76-2.2) | 4.02(3.46-4.67) |
|  | Hypophagia | 71 | 3.47(2.75-4.39) | 3.47(124.25) | 1.79(1.4-2.08) | 3.46(2.74-4.37) |
|  | Feeding disorder | 64 | 3.45(2.70-4.42) | 3.45(110.81) | 1.78(1.37-2.09) | 3.44(2.69-4.4) |
|  | Tumour lysis syndrome | 63 | 8.97(7.00-11.51) | 8.96(439.72) | 3.15(2.62-3.34) | 8.86(6.9-11.36) |
|  | Hypocalcaemia | 46 | 3.29(2.46-4.40) | 3.29(72.99) | 1.71(1.22-2.07) | 3.28(2.45-4.38) |
|  | Hyperammonaemia | 39 | 10.45(7.62-14.35) | 10.45(327.93) | 3.36(2.6-3.52) | 10.3(7.5-14.13) |
|  | Hypercalcaemia | 31 | 3.26(2.29-4.65) | 3.26(48.41) | 1.7(1.09-2.11) | 3.25(2.28-4.63) |
|  | Electrolyte imbalance | 29 | 3.37(2.34-4.85) | 3.37(47.97) | 1.75(1.11-2.16) | 3.35(2.33-4.83) |
|  | Malnutrition | 28 | 3.74(2.58-5.42) | 3.73(55.74) | 1.89(1.23-2.3) | 3.72(2.56-5.39) |
|  | Hypoalbuminaemia | 23 | 4.38(2.91-6.61) | 4.38(59.63) | 2.12(1.34-2.53) | 4.36(2.89-6.57) |
|  | Fluid intake reduced | 17 | 4.77(2.96-7.69) | 4.77(50.30) | 2.25(1.29-2.66) | 4.74(2.94-7.64) |
|  | Failure to thrive* | 15 | 4.53(2.73-7.53) | 4.53(40.96) | 2.17(1.16-2.61) | 4.5(2.71-7.48) |
|  | Hypoproteinaemia | 9 | 8.63(4.47-16.65) | 8.62(59.88) | 3.09(1.36-3.2) | 8.53(4.42-16.46) |
|  | Electrolyte depletion | 3 | 8.04(2.57-25.09) | 8.04(18.26) | 2.99(0.08-2.99) | 7.95(2.55-24.82) |
| Musculoskeletal and connective tissue disorders | Fistula | 65 | 8.03(6.28-10.25) | 8.02(394.54) | 2.99(2.49-3.2) | 7.93(6.21-10.13) |
|  | Myositis | 21 | 3.63(2.36-5.58) | 3.63(39.80) | 1.85(1.07-2.31) | 3.62(2.35-5.55) |
|  | Pathological fracture | 16 | 4.50(2.75-7.36) | 4.50(43.29) | 2.16(1.19-2.6) | 4.48(2.74-7.32) |
| Neoplasms benign, malignant and unspecified (incl cysts and polyps) | Malignant neoplasm progression | 1081 | 12.21(11.49-12.97) | 11.97(10687.67) | 3.56(3.45-3.63) | 11.77(11.07-12.51) |
|  | Tumour haemorrhage | 124 | 56.49(47.02-67.88) | 56.36(6207.70) | 5.7(4.94-5.47) | 51.96(43.25-62.44) |
|  | Liver carcinoma ruptured | 39 | 340.12(230.97-500.87) | 339.86(8668.90) | 7.81(4.56-5.62) | 223.93(152.07-329.77) |
|  | Cancer pain | 32 | 13.75(9.69-19.52) | 13.74(370.29) | 3.75(2.78-3.8) | 13.48(9.5-19.13) |
|  | Tumour pain | 27 | 21.53(14.67-31.59) | 21.52(511.47) | 4.38(3.05-4.16) | 20.87(14.22-30.62) |
|  | Tumour rupture | 22 | 50.83(32.94-78.44) | 50.81(996.74) | 5.56(3.35-4.59) | 47.22(30.59-72.87) |
|  | Malignant pleural effusion | 16 | 12.54(7.65-20.57) | 12.54(166.70) | 3.62(2.18-3.59) | 12.32(7.51-20.21) |
|  | Tumour necrosis | 10 | 12.62(6.75-23.60) | 12.62(104.94) | 3.63(1.73-3.48) | 12.4(6.63-23.18) |
|  | Tumour associated fever | 6 | 18.24(8.10-41.06) | 18.24(95.11) | 4.15(1.28-3.49) | 17.77(7.9-40) |
|  | Intracranial tumour haemorrhage | 5 | 16.02(6.60-38.91) | 16.02(68.72) | 3.97(0.99-3.38) | 15.66(6.45-38.03) |
|  | Haemorrhagic tumour necrosis | 3 | 140.06(40.25-487.40) | 140.05(341.09) | 6.85(0.37-3.56) | 115.51(33.19-401.98) |
|  | Infected neoplasm | 3 | 13.25(4.22-41.55) | 13.25(33.30) | 3.7(0.24-3.16) | 13(4.15-40.79) |
| Nervous system disorders | Hepatic encephalopathy | 418 | 63.19(57.13-69.89) | 62.67(23151.94) | 5.84(5.51-5.81) | 57.28(51.79-63.35) |
|  | Cerebral haemorrhage | 127 | 4.43(3.72-5.27) | 4.42(333.76) | 2.14(1.84-2.35) | 4.4(3.69-5.23) |
|  | Taste disorder | 108 | 6.41(5.30-7.75) | 6.40(487.54) | 2.67(2.32-2.88) | 6.35(5.25-7.67) |
|  | Cerebral infarction | 90 | 4.90(3.98-6.03) | 4.90(277.06) | 2.28(1.92-2.53) | 4.87(3.95-5.99) |
|  | Posterior reversible encephalopathy syndrome | 83 | 9.93(7.99-12.33) | 9.91(655.22) | 3.29(2.83-3.46) | 9.78(7.87-12.15) |
|  | Transient ischaemic attack | 64 | 3.00(2.34-3.83) | 2.99(84.63) | 1.58(1.18-1.89) | 2.98(2.33-3.82) |
|  | Encephalopathy | 51 | 2.85(2.16-3.75) | 2.85(60.93) | 1.51(1.05-1.86) | 2.84(2.16-3.74) |
|  | Cerebellar haemorrhage | 8 | 6.35(3.17-12.75) | 6.35(35.74) | 2.66(1.02-2.95) | 6.3(3.14-12.64) |
|  | Vocal cord paralysis | 7 | 5.33(2.53-11.22) | 5.33(24.44) | 2.41(0.76-2.81) | 5.3(2.52-11.15) |
|  | Hypertensive encephalopathy | 6 | 17.59(7.82-39.57) | 17.59(91.39) | 4.1(1.27-3.48) | 17.15(7.62-38.59) |
|  | Brain stem haemorrhage | 6 | 8.17(3.65-18.28) | 8.17(37.29) | 3.01(0.91-3.1) | 8.08(3.61-18.08) |
|  | Paralysis recurrent laryngeal nerve | 5 | 38.00(15.42-93.63) | 38.00(170.23) | 5.17(1.18-3.61) | 35.97(14.6-88.61) |
|  | Carotid artery perforation | 4 | 130.73(44.68-382.48) | 130.72(429.08) | 6.77(0.85-3.68) | 109.1(37.29-319.2) |
|  | Coma hepatic | 4 | 9.37(3.49-25.14) | 9.37(29.49) | 3.21(0.5-3.1) | 9.25(3.45-24.83) |
|  | Spinal cord haemorrhage | 3 | 8.45(2.71-26.40) | 8.45(19.46) | 3.06(0.1-3.01) | 8.36(2.68-26.1) |
|  | Thrombotic cerebral infarction | 3 | 8.34(2.67-26.06) | 8.34(19.15) | 3.04(0.1-3.01) | 8.25(2.64-25.77) |
|  | Limbic encephalitis* | 3 | 7.43(2.38-23.18) | 7.43(16.50) | 2.88(0.05-2.96) | 7.35(2.36-22.95) |
| Renal and urinary disorders | Proteinuria | 515 | 38.04(34.79-41.59) | 37.66(17383.57) | 5.16(4.93-5.19) | 35.67(32.62-38.99) |
|  | Renal impairment | 242 | 3.48(3.06-3.95) | 3.47(423.02) | 1.79(1.59-1.96) | 3.45(3.04-3.92) |
|  | Nephrotic syndrome | 60 | 12.42(9.62-16.03) | 12.40(617.30) | 3.61(2.99-3.74) | 12.19(9.44-15.74) |
|  | Nephritis | 13 | 4.95(2.87-8.54) | 4.95(40.65) | 2.3(1.17-2.72) | 4.92(2.85-8.49) |
|  | Urogenital fistula | 5 | 33.01(13.44-81.07) | 33.01(147.74) | 4.98(1.16-3.58) | 31.47(12.81-77.28) |
|  | Immune-mediated nephritis | 4 | 20.11(7.44-54.40) | 20.11(70.47) | 4.29(0.74-3.37) | 19.54(7.22-52.85) |
|  | Autoimmune nephritis | 4 | 15.56(5.77-41.95) | 15.56(53.24) | 3.93(0.67-3.29) | 15.22(5.65-41.04) |
| Reproductive system and breast disorders | Female genital tract fistula | 25 | 20.28(13.62-30.20) | 20.27(444.27) | 4.3(2.94-4.09) | 19.69(13.23-29.32) |
|  | Scrotal ulcer | 3 | 12.90(4.11-40.45) | 12.90(32.29) | 3.66(0.23-3.15) | 12.67(4.04-39.72) |
| Respiratory, thoracic and mediastinal disorders | Dysphonia | 459 | 9.62(8.77-10.56) | 9.55(3464.49) | 3.24(3.07-3.35) | 9.42(8.59-10.34) |
|  | Pulmonary embolism | 244 | 3.98(3.51-4.52) | 3.97(538.89) | 1.98(1.78-2.15) | 3.95(3.48-4.48) |
|  | Epistaxis | 240 | 3.73(3.28-4.23) | 3.71(473.54) | 1.89(1.68-2.06) | 3.7(3.26-4.2) |
|  | Interstitial lung disease* | 191 | 5.19(4.50-5.98) | 5.17(638.14) | 2.36(2.12-2.54) | 5.14(4.46-5.93) |
|  | Pleural effusion | 142 | 3.15(2.67-3.71) | 3.14(206.29) | 1.65(1.38-1.87) | 3.13(2.65-3.69) |
|  | Pneumothorax* | 124 | 9.81(8.21-11.71) | 9.79(964.04) | 3.27(2.91-3.44) | 9.66(8.09-11.53) |
|  | Haemoptysis | 119 | 5.25(4.39-6.29) | 5.24(405.59) | 2.38(2.07-2.6) | 5.21(4.35-6.24) |
|  | Pneumonitis | 92 | 4.17(3.40-5.12) | 4.17(220.23) | 2.05(1.7-2.3) | 4.15(3.38-5.09) |
|  | Tracheal fistula | 27 | 299.25(189.77-471.91) | 299.10(5503.25) | 7.68(4-5.25) | 205.51(130.32-324.07) |
|  | Pulmonary haemorrhage | 26 | 4.58(3.11-6.74) | 4.58(72.21) | 2.19(1.45-2.57) | 4.55(3.1-6.7) |
|  | Pharyngeal haemorrhage | 7 | 7.10(3.37-14.96) | 7.10(36.32) | 2.82(0.98-3.03) | 7.04(3.34-14.82) |
|  | Pneumothorax spontaneous* | 6 | 8.81(3.94-19.72) | 8.81(41.00) | 3.12(0.95-3.15) | 8.71(3.89-19.49) |
|  | Immune-mediated lung disease | 6 | 5.25(2.35-11.72) | 5.25(20.48) | 2.38(0.61-2.8) | 5.22(2.34-11.65) |
|  | Tracheal stenosis | 5 | 14.72(6.07-35.72) | 14.72(62.53) | 3.85(0.96-3.35) | 14.42(5.94-34.98) |
|  | Pulmonary artery thrombosis | 5 | 9.10(3.77-22.01) | 9.10(35.57) | 3.17(0.76-3.13) | 8.99(3.72-21.74) |
|  | Oropharyngeal fistula | 4 | 90.16(31.69-256.46) | 90.15(309.90) | 6.31(0.87-3.63) | 79.34(27.89-225.7) |
|  | Pulmonary cavitation* | 4 | 6.15(2.30-16.47) | 6.15(17.09) | 2.61(0.3-2.89) | 6.1(2.28-16.34) |
|  | Portopulmonary hypertension | 3 | 32.68(10.25-104.21) | 32.68(87.74) | 4.96(0.38-3.35) | 31.17(9.78-99.39) |
|  | Bronchial fistula | 3 | 20.43(6.47-64.46) | 20.42(53.74) | 4.31(0.32-3.27) | 19.84(6.29-62.59) |
|  | Lung perforation | 3 | 7.97(2.55-24.89) | 7.97(18.07) | 2.98(0.08-2.99) | 7.89(2.53-24.62) |
| Skin and subcutaneous tissue disorders | Palmar-plantar erythrodysaesthesia syndrome | 436 | 22.91(20.81-25.21) | 22.72(8751.13) | 4.46(4.25-4.53) | 21.99(19.98-24.2) |
|  | Skin ulcer | 82 | 3.86(3.11-4.80) | 3.86(172.63) | 1.94(1.57-2.21) | 3.84(3.09-4.77) |
|  | Hyperkeratosis | 33 | 7.55(5.36-10.65) | 7.55(185.37) | 2.9(2.15-3.15) | 7.47(5.3-10.54) |
|  | Erythema multiforme | 25 | 4.02(2.71-5.95) | 4.01(56.23) | 2(1.27-2.41) | 4(2.7-5.92) |
|  | Palmar erythema | 6 | 6.41(2.87-14.32) | 6.41(27.12) | 2.67(0.75-2.94) | 6.36(2.84-14.2) |
| Vascular disorders | Hypertension | 1765 | 11.36(10.83-11.92) | 11.00(15828.20) | 3.44(3.36-3.5) | 10.83(10.33-11.36) |
|  | Blood pressure fluctuation | 144 | 6.01(5.10-7.08) | 5.99(593.82) | 2.57(2.28-2.76) | 5.95(5.05-7.01) |
|  | Hypertensive crisis | 51 | 5.48(4.16-7.22) | 5.48(185.03) | 2.44(1.92-2.73) | 5.44(4.13-7.16) |
|  | Arterial haemorrhage | 30 | 31.90(22.11-46.03) | 31.88(855.67) | 4.93(3.43-4.49) | 30.45(21.1-43.93) |
|  | Aortic dissection | 23 | 5.20(3.45-7.84) | 5.20(77.33) | 2.37(1.55-2.73) | 5.16(3.43-7.78) |
|  | Embolism | 19 | 3.20(2.04-5.02) | 3.19(28.51) | 1.67(0.87-2.17) | 3.18(2.03-5) |
|  | Arterial rupture | 8 | 13.62(6.76-27.43) | 13.62(91.61) | 3.74(1.52-3.46) | 13.36(6.63-26.91) |
|  | Thrombophlebitis migrans | 7 | 17.27(8.15-36.57) | 17.26(104.50) | 4.07(1.46-3.53) | 16.85(7.95-35.68) |
|  | Artery dissection | 5 | 20.82(8.54-50.71) | 20.81(91.40) | 4.34(1.06-3.46) | 20.2(8.29-49.22) |
|  | Venous haemorrhage | 5 | 18.46(7.59-44.91) | 18.46(80.32) | 4.17(1.03-3.43) | 17.98(7.39-43.74) |
|  | Hypertensive urgency | 5 | 13.56(5.59-32.88) | 13.56(56.99) | 3.73(0.93-3.32) | 13.3(5.49-32.26) |
|  | Aortic thrombosis | 5 | 6.50(2.69-15.68) | 6.50(23.03) | 2.69(0.57-2.94) | 6.44(2.67-15.55) |

*Emerging findings of lenvatinib associated AEs from FAERS database. ROR, reporting odds ratio; CI, confidence interval; PRR, proportional reporting ratio; χ2, chi-squared; IC, information component; EBGM, empirical Bayesian geometric mean.
